# Endoscopic biliary drainage for malignant distal biliary obstruction: Which is better – endoscopic retrograde cholangiopancreatography or endoscopic ultrasound?

**DOI:** 10.1111/den.14186

**Published:** 2021-11-29

**Authors:** Ji Young Bang, Robert Hawes, Shyam Varadarajulu

**Affiliations:** ^1^ Digestive Health Institute Orlando Health Orlando USA

**Keywords:** cholangiocarcinoma, ERCP, EUS, malignant distal biliary obstruction, pancreatic cancer

## Abstract

Presently, following endoscopic ultrasound (EUS)‐guided biopsy, an endoscopic retrograde cholangiopancreatography (ERCP) with transpapillary stenting is performed for palliation of malignant distal biliary obstruction (MDBO). However, technical failure and postprocedure pancreatitis are limitations to ERCP. Endoscopic ultrasonography‐guided biliary drainage (EUS‐BD) after a failed ERCP has a 90% technical success rate and has been shown to be superior when compared to percutaneous methods, making EUS an increasingly recognized option for biliary drainage. Supporting this approach, findings from recently concluded randomized trials suggest that the safety profile and technical outcomes for EUS‐BD are comparable or even superior to that of ERCP for primary biliary decompression in patients with MDBO. Also, EUS‐BD is increasingly being utilized in patients with altered surgical anatomy in lieu of percutaneous techniques and balloon‐assisted enteroscopy. A growing body of evidence supports the notion that, in the future, EUS may become the primary modality by which biliary decompression is undertaken in the majority of patients with MDBO. The roadmap to this eventuality may require further optimization of procedural techniques, technological innovations, and cost reduction.

## INTRODUCTION

Although endoscopic retrograde cholangiopancreatography (ERCP) is the primary technique to access the bile duct to undertake therapeutic interventions, the first essential procedural step, biliary cannulation, can fail in 0.5–16% of procedures in expert hands and may be even higher for novice endoscopists.^1^, ^2^, ^3^ When standard ERCP cannulation maneuvers fail, advanced cannulation techniques or precut (access) sphincterotomy or fistulotomy may be required to gain biliary access. However, adverse events have been reported in 2–34% of ERCP procedures performed using precut techniques, a rate that is significantly higher than that reported for standard cannulation techniques.^4^, ^5^, ^6^ Endoscopic ultrasound (EUS) is a highly sensitive and popular modality for diagnosing pancreatic cancer. In patients with malignant distal biliary obstruction (MDBO), the dilated extrahepatic bile duct can be imaged with relative ease when the echoendoscope is positioned in the duodenal bulb. The anatomic window provides an ideal access route to perform EUS‐BD (biliary drainage) via transduodenal stent placement (choledochoduodenostomy). When endoscopic access to the duodenum is anatomically impeded, biliary drainage can be undertaken via the stomach by placement of a self‐expandable metal stent (SEMS) in the dilated intrahepatic bile duct (hepaticogastrostomy) or by advancing the endoprosthesis through the ampullary orifice (antegrade stenting). Although these methods have traditionally been used only as a rescue measure after failed ERCP, randomized trials have demonstrated high rates of technical success and an acceptable safety profile comparable to ERCP when performed as the first‐line treatment measure.^7^, ^8^, ^9^ Additionally, observational studies have shown that, using EUS, biliary decompression can be achieved successfully in greater than 90% of patients who have failed prior attempts at ERCP.^10^, ^11^ Consequently, the logical question is whether ERCP or EUS is better as the primary technique for establishing drainage in MDBO? This review examines the currently available data, existing procedural limitations, implications for training, and draws a roadmap for the future.

## Present status of ERCP

**Figure 1:**
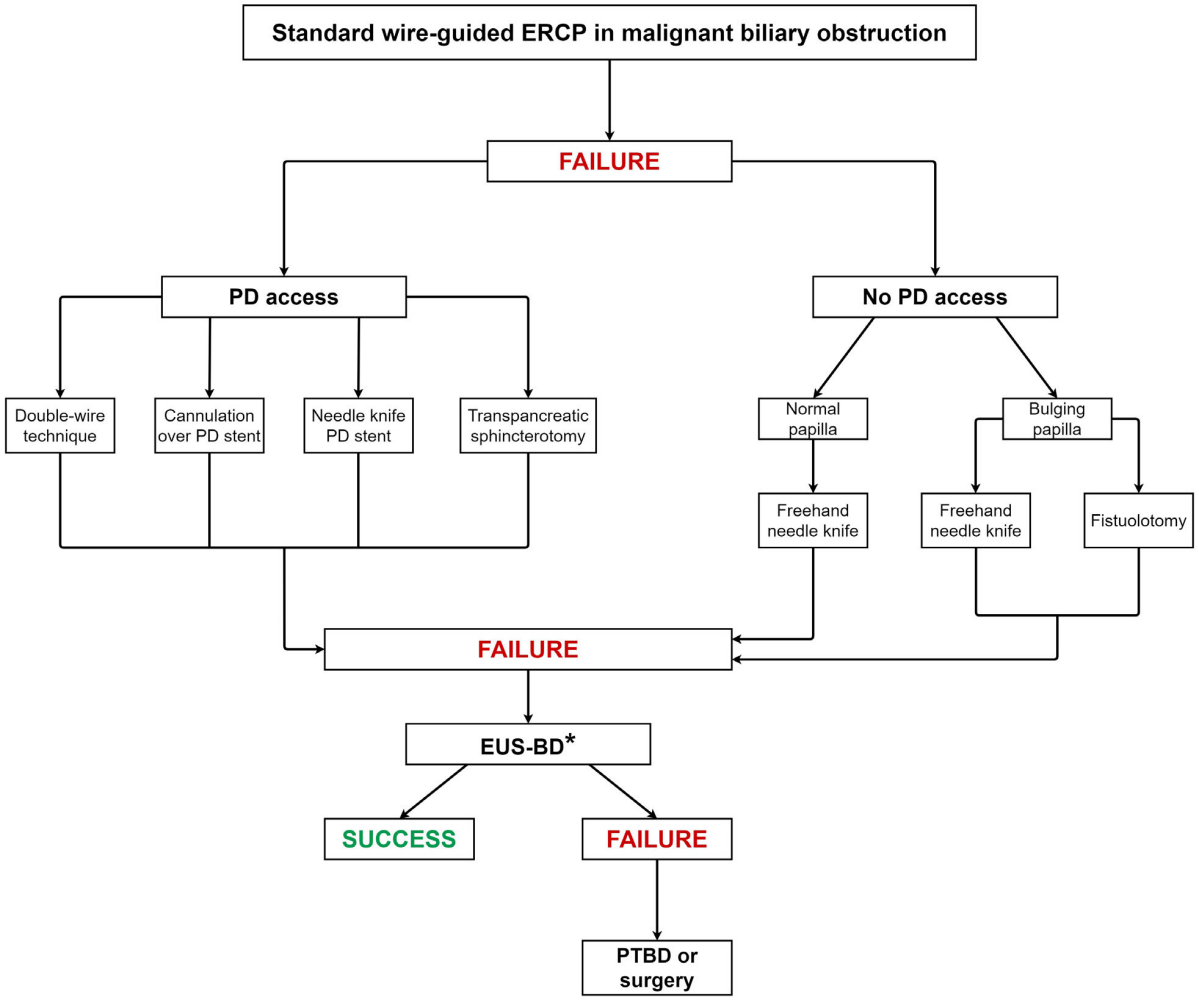
Stepwise approach to biliary cannulation at endoscopic retrograde cholangiopancreatography. *If endoscopic ultrasonography‐guided biliary drainage (EUS‐BD) is not available, patient may directly undergo percutaneous transhepatic biliary drainage (PTBD).

The international consensus statement for management of malignant distal biliary stricture recommends ERCP with transpapillary SEMS placement as the mainstay of treatment.^12^ In clinical practice, the three commonly encountered challenges with this recommendation are cannulation difficulties, adverse events, and stent dysfunction. Cannulation in patients with distal biliary obstruction can be challenging because of edema and friability in the second portion of the duodenum, distortion of the ampulla due to tumor infiltration, or duodenal narrowing limiting maneuverability. One meta‐analysis reported that 25–45% of patients undergoing ERCP for MDBO were observed to have some degree of duodenal involvement by the tumor.^13^ Distorted papillary anatomy may require advanced cannulation techniques, such as precut sphincterotomy, to gain ductal access and such maneuvers can be associated with significant rates of adverse events that include pancreatitis, bleeding, and perforation.^14^ It is well established that there is a direct correlation between procedural complexity and adverse events, particularly pancreatitis: rates of post‐ERCP pancreatitis are <3% if cannulation is achieved within 5 min versus >10% if it takes more than 10 min to achieve cannulation or with more than 10 cannulation attempts.^15^, ^16^ However, when ERCP is performed at tertiary referral centers by expert endoscopists, the outcomes are optimal. In a prospective study we observed that selective cannulation can be achieved in 99.4% of patients and requisite therapy such as endoprosthesis placement may be performed successfully when the ampulla is endoscopically accessible.^1^ Figure 1 shows a stepwise approach to biliary access adopting ERCP techniques.

Despite successful intervention, stent dysfunction, due to either occlusion by tumor or endoprosthesis migration, is a potentially serious delayed adverse event that can result in life‐threatening cholangitis. Although covered‐SEMS have been developed with the objective of improving stent patency, those expectations have been met with only partial success.^17^, ^18^ Other limitations associated with the use of covered‐SEMS include stent migration and cholecystitis in patients with gallbladder in‐situ.

From a training perspective, a recent study demonstrated that only 60% of advanced endoscopy fellows achieve technical competence in basic ERCP skills at completion of a fellowship.^19^ The learning curve for advanced techniques such as precut sphincterotomy is steep, and improves with growing experience.^20^

## Present status of EUS‐BD

The international consensus statement for management of malignant distal biliary stricture recommends that, when expertise is available, EUS‐BD may be an effective option in three situations: failed ERCP, difficult biliary cannulation, and postsurgical anatomy.^12^ Although percutaneous techniques have long been utilized in these situations, a randomized trial observed that EUS‐BD was associated with lower rates of adverse events (8.8 vs. 31.2%, *P* = 0.022) and fewer unscheduled reinterventions (0.34 vs. 0.93, *P* = 0.02) when compared to percutaneous methods.^21^ These findings were subsequently validated in a meta‐analysis that included 16 studies and 528 patients.^22^ EUS‐BD may be categorized according to the route of approach and the site of biliary drainage: choledochoduodenostomy, hepaticogastrostomy, rendezvous technique, and antegrade biliary stenting. As the rendezvous technique essentially provides only a guidewire access to undertake ERCP, this technique will not be discussed in this review.

Accessing the papilla at endoscopy may be difficult or impossible in patients with duodenal stenosis or surgically altered anatomy. The success rate of ERCP in these situations is dependent on reaching the papilla, which may not be possible in up to 40% of patients.^23^ EUS‐BD is an effective alternative in such situations, as the bile duct can be accessed from the proximal stomach or via the duodenal bulb. A systematic review of 42 studies with 1192 patients who underwent EUS‐BD after a failed ERCP reported a technical success rate of 94.7%, clinical success rate of 91.6%, and adverse event rate of 23.32%, which included bleeding (4.03%), bile leakage (4.03%), pneumoperitoneum (3.02%), stent migration (2.68%), cholangitis (2.43%), abdominal pain (1.51%), and peritonitis (1.26%).^24^ A meta‐analysis of three recent randomized trials has shown that EUS‐BD is comparable to ERCP in terms of technical and clinical outcomes when used as the primary treatment measure for MDBO.^13^ While there are no data from prospective or randomized clinical trials, retrospective studies have shown that EUS‐guided techniques can establish biliary drainage in patients with altered surgical anatomy via antegrade stenting, transgastric/duodenal routes, or EUS‐directed transgastric ERCP. A recent review reported an overall technical success rate of more than 90% and adverse event rates between 10% to 20% for an EUS‐based treatment approach in this patient cohort.^25^

In terms of access, the biliary system can be accessed via the transduodenal and transgastric routes. Both approaches have been found to be effective, provided the ducts are adequately dilated. There is a lack of clarity about the preferred route when both approaches are technically feasible. Current data are conflicting, with some reports showing the transduodenal route to be safer, while others demonstrated no such difference.^26^, ^27^ In a small randomized study comparing 25 patients who received hepatogastrostomy and 24 who received choledochoduodenostomy, the clinical success for hepatogastrostomy was higher (91% vs. 77%); however, the adverse events were also slightly higher (20% vs. 12.5%), although neither outcome reached statistical significance.^28^ There is no prospective data comparing the performance of antegrade stenting with other techniques. Thus, in patients with MDBO, any of these approaches could be performed, with the choice based on a combination of factors including procedural expertise, risk of adverse events, and anatomical factors such as the presence of dilated bile duct or biliary radicals, duodenal stenosis, and altered anatomy. While fully or partially covered SEMS are recommended for transluminal stenting, uncovered metal stents can be used for antegrade transpapillary stenting.^29^

**Figure 2:**
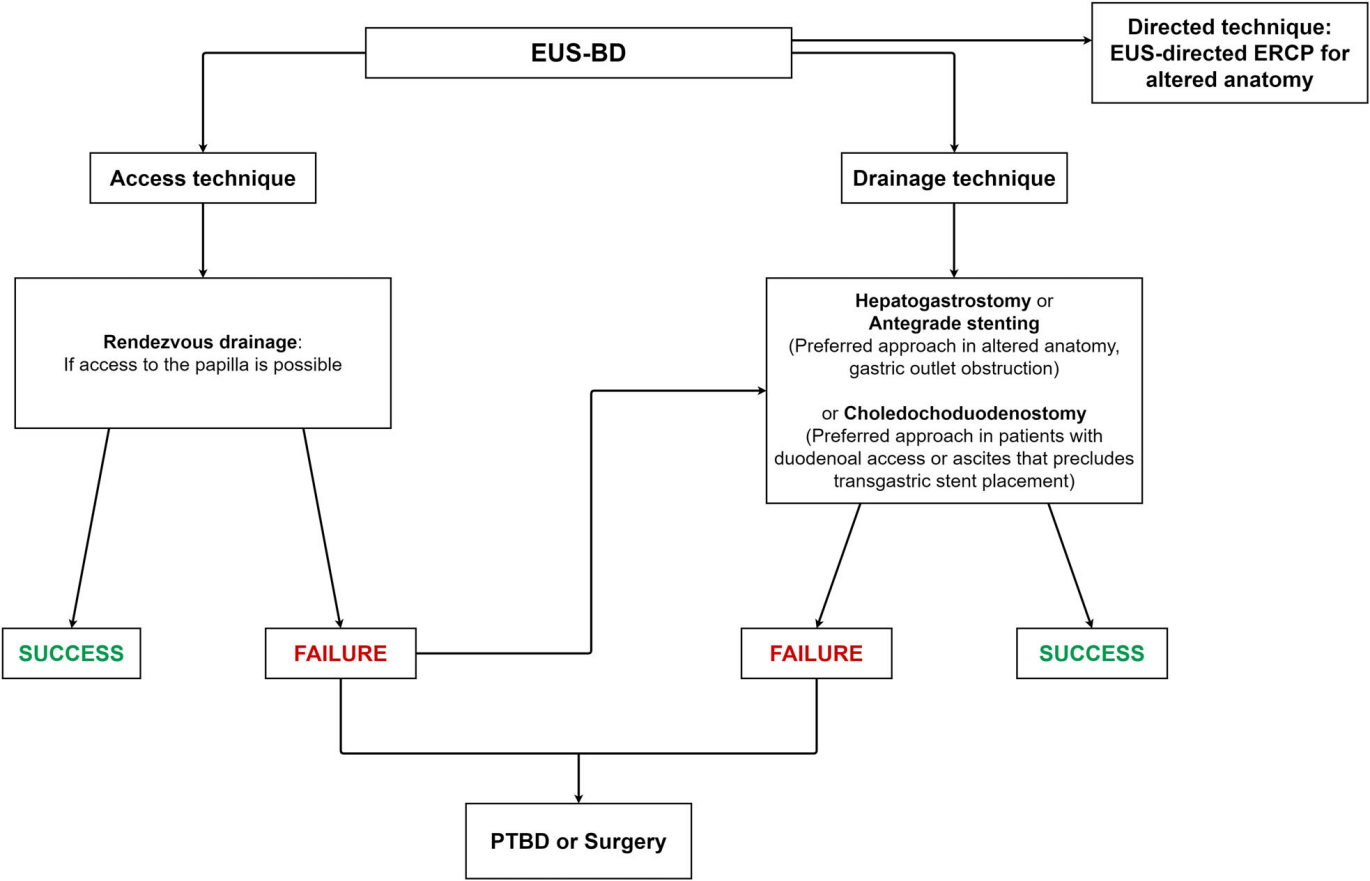
Stepwise approach to biliary access adopting endoscopic ultrasonography‐based techniques.

Recently, short dumbbell‐shaped fully covered lumen‐apposing metal stents (LAMS) have been developed for choledochoduodenostomy (Hot AXIOS; Boston Scientific, Marlborough, MA, USA). These stents are made available in small‐size diameters (6, 8 mm) in hopes of minimizing adverse events such as leak. The main advantage is that their deployment is a single‐step process that significantly shortens procedural time. A recent meta‐analysis examined seven studies including 284 patients who underwent EUS‐BD using LAMS after a failed ERCP.^29^ The pooled rates of technical and clinical success were 95.7% and 95.9%, respectively. The pooled rate of postprocedure adverse events was 5.2% and recurrent jaundice was observed in 8.7% of patients. Recurrent jaundice was mostly due to stent occlusion from sludge or debris and stent migration. A major limitation of EUS‐BD is that the procedure can be undertaken only when the biliary ductal system is dilated, particularly when LAMS placement is contemplated. Figure 2 shows a stepwise approach to biliary access adopting EUS‐based techniques.

EUS‐BD is an advanced endoscopic technique that shares similar skillsets and accessories with ERCP, but unlike ERCP there are no formal training programs specific to the technique. A recent study demonstrated that 82% of advanced endoscopy fellows achieve technical competence in EUS at conclusion of the fellowship.^19^

## ERCP versus EUS‐BD: WHICH IS BETTER?

To answer this, three fundamental questions need to be addressed.

- Optimal clinical outcome – which technique will yield the highest technical and clinical success but with minimal adverse events in the majority of patients with MDBO?
- Roadmap to future – what limitations, if any, must be overcome to achieve the most optimal clinical outcome?
- Training – what is required to train the next generation of endoscopists in state‐of‐the‐art procedural techniques?

### Clinical outcome

Based on the results of a meta‐analysis that included three randomized trials and two observational studies that compared ERCP and EUS‐BD for primary biliary decompression, both techniques were found to be equally effective in achieving biliary drainage (ERCP = 94.73%; EUS = 93.67; pooled odds ratio [OR] 1.20; 95% confidence interval [CI] 0.44–3.24) and resolving jaundice (ERCP = 94.21%; EUS = 91.23%; pooled OR 1.44; 95% CI 0.63–3.29).^13^ While there was also no significant difference in the overall rate of procedure‐related adverse events (ERCP = 22.3%; EUS = 15.2%; OR 1.59; 95% CI 0.89–2.84), postprocedure pancreatitis was significantly higher for ERCP (9.5% vs. EUS = 0; risk difference 8%; 95% CI 1–14%). There was no significant difference in rates of reinterventions for jaundice between groups (ERCP = 22.6%; EUS = 15.2%; OR 1.68; 95% CI 0.76–3.73), which were mostly due to tumor overgrowth/ingrowth for the ERCP group and due to food debris or sludge for EUS. Another meta‐analysis reported similar outcomes with no differences in reinterventions, procedure duration, stent patency, and overall survival between cohorts.^30^ When compared to ERCP, the bulk of data suggests that the performance of EUS‐BD is equivalent for primary biliary decompression. However, EUS‐BD is superior to percutaneous methods as a rescue measure after failed ERCP and appears indispensable for establishing biliary drainage in patients with altered surgical anatomy. Similar to percutaneous methods, EUS is unlikely to be successful if the biliary ductal system is not dilated. A caveat is that almost all data pertaining to EUS‐BD originated from highly specialized centers with procedures being performed by expert endosonographers.

#### Bottom line

One can conclude that EUS‐BD yields the highest technical success when compared to ERCP in the majority of patients with MDBO and with a comparable safety profile. The most common adverse event of ERCP, postprocedure pancreatitis, is minimal with EUS‐BD.

### Roadmap for the future

The procedural technique for ERCP is well standardized. It involves access (deep cannulation) followed by sphincterotomy and stent placement. If ductal access is unsuccessful, then alternate methods are required to establish biliary drainage. On the other hand, accessing a dilated biliary ductal system at EUS is technically easy, but subsequent steps such as transmural tract dilation and placing a stent in the correct axis can be challenging. Given the multiple methods that are currently practiced at EUS to decompress the bile duct, the procedural techniques have not been well standardized. A majority of studies on MDBO have focused on choledochoduodenostomy, followed by hepatogastrostomy, and then antegrade stent placement. In a meta‐analysis that included 10 studies comparing choledochoduodenostomy and hepatogastrostomy, the pooled odds ratio for rates of technical success, clinical success, and adverse events were 1.36 (95% CI 0.66–2.81; *P >* 0.05), 0.84 (95% CI 0.50–1.42; *P >* 0.05), and 0.61 (95% CI 0.36–1.03; *P >* 0.05), respectively, which indicated no significant difference between the two groups.^24^ While some of the studies utilized cautery devices for transmural tract puncture and dilation, others used small‐caliber dilators to facilitate faster healing of the puncture site and thereby reduce bile leak. Give the small number of patients, there are no comparative data between antegrade stenting and other methods.

Due to the lack of technical standardization, the development of accessories specific to EUS‐BD has been slow. While dedicated LAMS have been developed and are currently being used to perform choledochoduodenostomy, their performance has been less than stellar.^29^ As the endoprosthesis are deployed proximal to the tumor margins, it has been postulated that, unlike transpapillary stent placement, stents placed at EUS‐BD may have longer patency and therefore less need for reinterventions. Unfortunately, it has been observed that 8.7–15.2% of patients may require additional interventions due to recurrence of jaundice or, rarely, stent migration.^13^, ^29^ It is postulated that the distal flange of the dumbbell‐shaped LAMS, on biliary decompression, may reside horizontally in the axis of the duct, resulting in stagnation of bile. To overcome this limitation, some experts have recommended placement of a long double‐pigtail plastic stent within the LAMS so as alter its orientation and augment biliary drainage.^31^ Other specially designed SEMS and plastic stents have been proposed for use in hepatogastrostomy.^32^, ^33^, ^34^ However, clinical experience with these stents are limited, and they are not widely available in many parts of the world.

Unlike ERCP, EUS‐BD has multiple access points to the biliary ductal system from the gastrointestinal lumen. The procedural technique variants therefore may require tailoring to meet individual patient needs. Given the promise that EUS‐BD holds for the future, it is important to develop a well‐thought‐out roadmap – it would perhaps make sense to first focus on the more widely practiced technique of choledochoduodenostomy followed by hepatogastrostomy. This would include steps such as identifying safe methods for transmural tract dilation (cautery vs. dilators), developing dedicated devices to access nondilated biliary ductal systems and for steering guidewires in the desired direction, constructing less expensive single‐step device platforms, and dedicated stents with longer patency. Until such time, given the lack of standardization of EUS‐BD and the potential risk for serious adverse events, ERCP and percutaneous transhepatic biliary drainage should be the standard of care when tertiary‐level expertise in EUS‐BD is not available.

#### Bottom line

**Figure 3:**
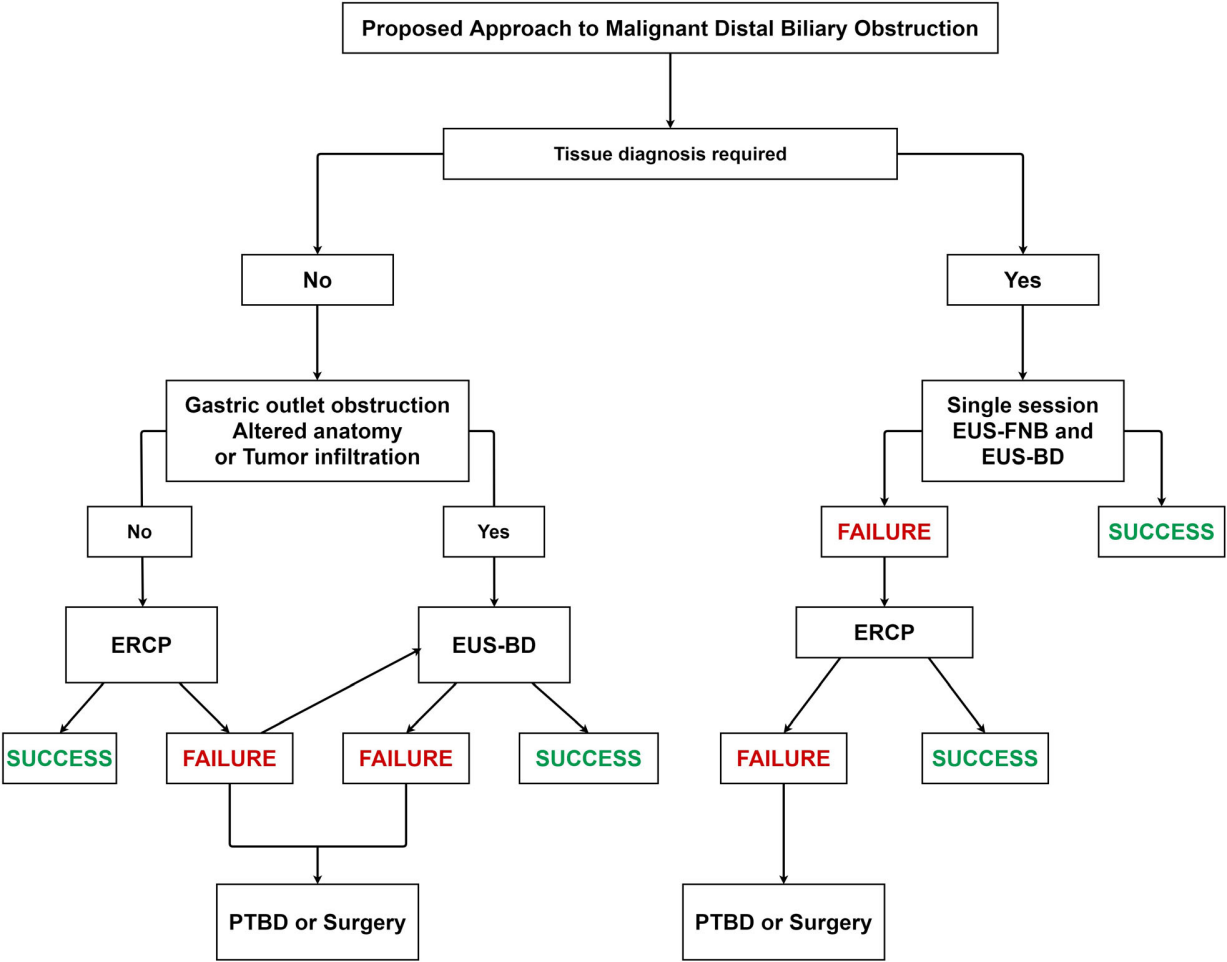
Proposed future approach to endoscopic biliary drainage.

It is obvious that while EUS‐BD can be performed effectively and efficiently in expert hands, further procedural standardization and technological refinements are required to facilitate widespread adoption. We propose an algorithm for futuristic adoption of EUS‐BD that may be currently applicable only in centers where technical expertise, access to technology, and multidisciplinary support is available (Fig. 3).

### Training

More trainees achieve technical competence in basics of EUS than ERCP at completion of advanced fellowship training.^19^ This is likely because, unlike EUS, ERCP is a purely therapeutic procedure in which practical experience with complex techniques such as access sphincterotomy are limited. It is also more challenging than other endoscopic procedures because it requires excellent hand–eye coordination and full command of multiple maneuvers to find the optimal angles to achieve deep cannulation. The longer the endoscopist takes to cannulate, the greater is the risk of pancreatitis and the need for pancreatic stenting. On the other hand, EUS‐BD only requires finding a window to the chosen section of the bile duct. Intuitively, an obstructed/dilated bile duct can be more easily visualized and accessed at EUS than by performing a semi‐blind maneuver such as precut sphincterotomy at ERCP. If cannulation fails at ERCP, the patient is obligated to undergo drainage under EUS or percutaneous guidance; but if ductal access cannot be achieved using a particular technique at EUS, other methods can be adopted in the same session to achieve the objective. Given these advantages, it is essential to impart training in the different components of EUS‐BD such as fine‐needle aspiration, wire manipulation, and endoprosthesis deployment, all of which are performed more under sonographic and radiologic visualization than an endoscopic view. This may require training in models and serving as operator or assistant during ERCP procedures to gain familiarity with the procedural steps prior to hands‐on experience in patients. Table 1 is a take‐home message on treatment of malignant distal biliary decompression using endoscopic techniques.

**Table 1:** Take‐home message

EUS‐BD yields the highest technical success when compared to ERCP in the majority of patients with MDBO and with a comparable safety profile. The most common adverse event of ERCP, postprocedure pancreatitis, is minimal with EUS‐BD. While it is obvious that EUS‐BD can be performed effectively and efficiently in expert hands, further procedural standardization and technological refinements are required to facilitate widespread adoption. We propose an algorithm for futuristic adoption of EUS‐BD. Training in EUS‐BD is of paramount importance, given the promising future potential for this treatment approach. Development of a curriculum that incorporates training in models, observation at expert centers, gaining proficiency in individual procedural components, and hands‐on training with proctoring of complex cases is required to advance the practice.

ERCP, endoscopic retrograde cholangiopancreatography; EUS, endoscopic ultrasound; EUS‐BD, endoscopic ultrasonography‐guided biliary drainage; MDBO, malignant distal biliary obstruction.

#### Bottom line

Training in EUS‐BD is of paramount importance, given the promising future potential for this treatment approach. Development of a curriculum that incorporates training in models, observation at expert centers, gaining proficiency in individual procedural components, and hands‐on training with proctoring of complex cases is required to advance the practice.

Finally, cost analysis is an important component of any future advancement. Although EUS‐BD is significantly less costly than percutaneous techniques, there are no financial data comparing EUS‐ and ERCP‐based methods for primary biliary decompression.^35^ Simplification of the procedural technique and development of less costly single‐step devices may be required to achieve this goal.

## CONCLUSION

Endoscopic ultrasound is as safe and effective as ERCP for achieving palliation in patients with malignant distal biliary obstruction. The technique is particularly useful in patients with surgically altered anatomy and those in whom access to the major duodenal papilla is limited. Additional technological innovations, technical refinements, and development of training platforms can accelerate its widespread adoption. We foresee the possibility of EUS‐BD being the primary treatment measure for patients with MDBO in the future. However, given that EUS is a relatively new technology, more endoscopists worldwide have had greater exposure to ERCP than EUS. Also, ERCP is less resource‐consuming and is more widely available globally. Therefore, the transition from ERCP to EUS‐BD will take time for this gradual “generational” change to occur.

## Conflict of Interest

J.Y.B. is a consultant for Boston Scientific, Olympus, Fujifilm Medical Systems. R.H. is a consultant for Olympus. S.V. is a consultant for Boston Scientific, Olympus, Medtronic.

## Funding Information

None.
